# Bignoniaceae Metabolites as Semiochemicals

**DOI:** 10.3390/molecules15107090

**Published:** 2010-10-14

**Authors:** Lucía Castillo, Carmen Rossini

**Affiliations:** Laboratorio de Ecología Química, Facultad de Química, UdelaR, Gral. Flores 2124, Montevideo, CP 11880, Uruguay; E-Mail: lcastillo@fq.edu.uy (L.C.)

**Keywords:** kairomones, allomones, semiochemicals, Bignoniaceae, insect-plant interactions, iridoids, quinones, *Epilachna*, *Spodoptera*, *Myzus*, *Rhopalosiphum*, *Boophilus*, deterrent, phagostimulant

## Abstract

Members of the family Bignoniaceae are mostly found in tropical and neo-tropical regions in America, Asia and Africa, although some of them are cultivated in other regions as ornamentals. Species belonging to this family have been extensively studied in regard to their pharmacological properties (as extracts and isolated compounds). The aim of this review is to summarize the reported scientific evidence about the chemical properties as well as that of the extracts and isolated compounds from species of this family, focusing mainly in insect-plant interactions. As it is known, this family is recognized for the presence of iridoids which are markers of oviposition and feeding preference to species which have became specialist feeders. Some herbivore species have also evolved to the point of been able to sequester iridoids and use them as defenses against their predators. However, iridoids also exhibit anti-insect properties, and therefore they may be good lead molecules to develop botanical pesticides. Other secondary metabolites, such as quinones, and whole extracts have also shown potential as anti-insect agents.

## 1. Introduction

The family Bignoniaceae (order Lamiales) includes 120 genera with 800 species mainly distributed within tropical and neo-tropical regions of America, Asia and Africa; however, some species are also used worldwide as ornamentals [1]. 

Bignoniaceae species have been studied in regard to the ecological and evolutionary roles of their secondary metabolites that mediate interactions among plants and their herbivores. The family is recognized for the presence of iridoids [2], bitter compounds which not only exhibit anti-insect properties, but also are oviposition and feeding stimulants for specialist species [3,4]. In addition, some specialist species have evolved the capacity to sequester iridoids and use them as defenses against their predators either as such or after bio-transformation [5]. Besides, several works have reported on the ethnobotanical uses of both complete extracts and isolated secondary metabolites (other than iridoids) from Bignoniaceae. These uses comprise from applications as insect repellents (*i.e*. *Mansoa *sp. extracts) to systemic utilization (*i.e*. *Tecoma stans* infusions used as antidiabetic) [6,7,8]. Particular groups of natural products from Bignoniaceae have been shown to have potential healing uses, such as antimicrobial activity (*i.e.* anthraquinones, flavonoids, phenylpropanoid glycosides isolated from members of the genera *Tabebuia* and *Arrabaidea* [7,9,10,11,12,13]), and anti-parasitic activity (*i.e*. anti-malarial naphthoquinones isolated from the barks of *Sterespermum kunthianum* [14] and *Tabebuia incana* [15]). Indeed, studies of anti-parasitic properties have been driven by the overlap of Bignoniaceae world distribution and the incidence of parasitic diseases. Among naphthoquinones, lapachol, isolated first from *Tabebuia avellandeae* trees, has numerous biological activities [16]. Lapachol capacity as an anti-malarial and anti-leishmania agent has driven further studies of derivatives and analogues [17,18]. All these pieces of information have been previously reviewed [1,6,7,8,9,10,11,12,13].

Since the seminal work from Erlich and Raven [19], the work on natural product chemistry has drifted from merely the reports on structural information of secondary metabolites to the description that also accounts for the ecological interactions mediated by those secondary metabolites. At the same time, the huge development of the field of Chemical Ecology [20,21], lead to a whole new approach of looking at natural products as mediating many of the interactions among leaving beings. New terms have been coined for secondary metabolites emphasizing their role as signal bearing a specific message. In this way, semiochemicals (from Greek *semeion* = sign) are recognized as chemical signs able to modify either particular behaviors (releasers), or physiological processes (primers) [22]. Among semiochemicals, two groups can be recognized: first, pheromones which mediate interactions between members of the same species [23]; and second allelochemicals which ascertain relations between organisms belonging to different species. Among allelochemicals, signals can be classified according to the benefit they provide to the sender (producer of the secondary metabolite) and to the receiver organism. In this manner, allomones (advantageous to the sender), kairomones (advantageous to the receiver) and synomones (advantageous to both) establish different interactions [22]. 

The following appraisal will examine the available information on extracts and secondary metabolites from Bignoniaceae, under the light of these definitions of semiochemicals, with emphasis on works investigating insect-plant interactions. Further, since allomones have evolved in the case of plants under the evolutive pressure of herbivores (and other etiological agents of various pathologies) [19] we shall also present a compilation of potential anti-insect agents from Bignoniaceae to be used against pests. 

## 2. Iridoids

**Figure 1:**
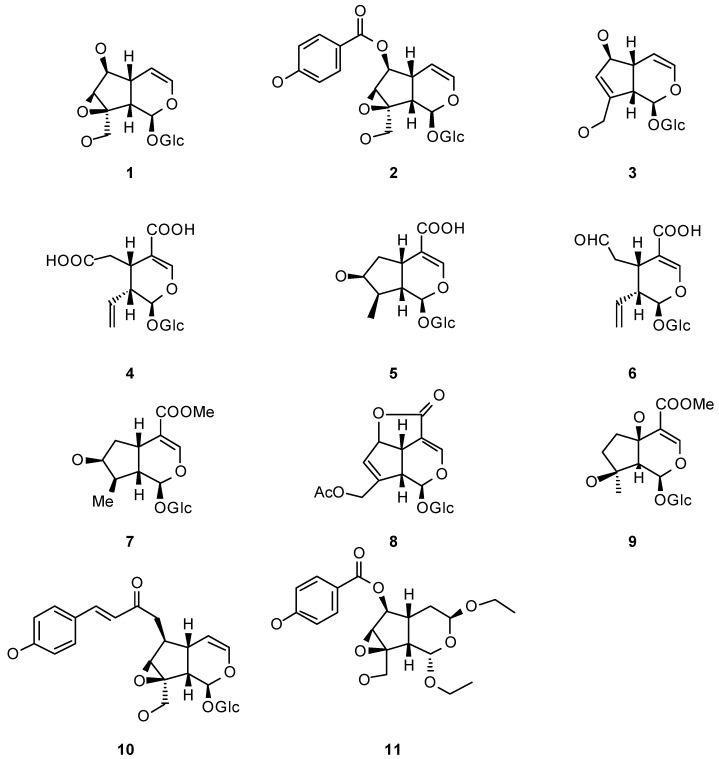
Examples of Bignoniaciae iridiods.

The presence of iridoids (*i.e. ***1-11**, Figure 1) is characteristic of several tribes within Bignoniaceae. These compounds, as cyclopentane monoterpenes, are biosynthesized *via* the mevalonic acid pathway, and were first isolated from *Iridomyrmex* ants as defensive compounds. Iridoids occurred in about 57 families of plants, including Bignoniaceae [24].

Catalpol (**1**), isolated from *Catalpa* species and probably the most representative iridoid, and related iridoids have been extensively studied for their ecological relevance. Originally considered to be defensive compounds, these iridoids have been tested in many different anti-insect capacities. However, several studies have shown that specialist insects are able to use them as semiochemicals, such as kairomones and allomones. 

### 2.1. Iridoids as Markers for Food Choice

The interaction between the moth *Ceratomia catalpae* (Lepidoptera: Sphingidae; from now on abbreviated *Ce. catalpae*) and its food plants, species belonging to the genus *Catalpa*, provides a good example of the role of iridoids as chemical clues during the whole life cycle of a specialist insect. 

Food choice plays a central role in the appropriate development of herbivore Lepidoptera. This selection of the suitable diet can be done by adults, when they select where to lay eggs or by larvae when they wonder from plant to plant later in the life cycle (if the previous adult decision was poor [25,26]). Both decisions are often determined by semiochemicals (oviposition stimulants and phagostimulants respectively), usually of the kairomone kind. 

Iridoids may contribute to the relation established between *Ce. catalpae* and its food plants. In laboratory assays, when catalpol and catalposide (**2**) were offered in sucrose solutions to *Ce. catalpae*, these larvae preferred those solutions instead of the iridoid-free controls [27]. This observation probably indicates that in Nature, iridoids are also phagostimulants to *Ce. catalpae *larvae which feed on leaves that contain iridoids. Likewise, catalpol and catalposide are also present in nectar from *Catalpa speciosa *flowers, where they may play some ecological role, but not as pollinator markers to *Ce. catalpae* larvae, which are considered not to be good pollinators in this system [28]. Pollination in *C. speciosa* is dual [28]: diurnal by carpenter ants and small bees and nocturnal by moths including sphyngids among which *Ce. catalpae* adult may be expected to be [29]. In this regard, it would be interesting to run preference bioassays for adults similar to the ones run for larvae [27], to answer if in this context, iridoids may be working as pollination synomones. These studies may contribute to the understanding of the interactions between both species. Iridoid biosynthesis in *Catalpa *spp. may be in the process of being selected against as phagostimulants to larvae as they do not provide a benefit to the plant when accomplishing this role (kairomones). On the other hand, there is a chance that a positive selective pressure on *Catalpa *spp. for keeping iridoids may come from the fact that these semiochemicals constitute pollination clues to the adults (synomones). Besides, it is known that *Catalpa *spp. are visited by nectar thieves [27]. In this line, iridoids were shown to be deterrents to small ants and skippers which preferred iridoid-free solutions of sucrose rather than iridoid-loaded solutions or the nectar itself. Further, if consumed, iridoids brought about different sorts of behavioral abnormalities to both kind of nectar thieves [27]. In the interaction between nectar thieves and *Catalpa* spp., iridoids are therefore beneficial to the plant, working as an effective defense (allomones) for *C. speciosa *(in this particular study). Therefore the cost of having iridoids to *Catalpa *spp. in regard to herbivory may be overcome by the benefits when they act as effective defenses or pollination facilitators.

In the system *Ce. catalpae*-*Catalpa *spp., one issue that remains to be explored is whether iridoids also act as oviposition stimulants (a function that have been described for iridoids in other plant families). *Plantago lanceolata* (Plantaginaceae) iridoids are markers to *Melitaea cinxia *and *Junonia coenia* (Lepidoptera: Nymphalidae) adults which prefer to oviposit on leaves with higher contents of catalpol and aucubin (**3**) [30]. However, not all Lepidoptera specialists on iridoid-containing plants behave similarly. Another nymphalid, *Euphydryas aurinia* which feeds on *Lonicera implexa* (Caprifoliaceae) leaves containing secoxyloganic acid (**4**), loganic acid (**5**) and secologanic acid (**6**), does not use them as oviposition stimulants [31], but rather, after eggs are laid on the leaves, the plant respond dramatically increasing local amounts of these iridoids (see below under induction).

### 2.2. Fate of Iridoids after Consumption

Once consumed, iridoids from *Catalpa* spp. are managed by *Ce. catalpae *larvae in different ways. The fate of catalpol (**1**) and its ester, catalposide (**2**) is different. Even though plants produce eight times less catalpol than catalposide, the former is the only one sequestered by larvae. Authors hypothesized that this selectivity araises from the catalposide hydrolysis in the larva gut before being assimilated as catalpol. Besides, some other forms of systemic metabolization or elimination must be present in the larvae, as catalpol amount declines trough the life cycle (in adulthood, catalpol is not longer detected). The not yet assimilated iridoids are present in the reflex regurgitate liberated after larva disturbance, probably as a chemical defense [5,32]. It remains to be experimentally probed whether iridoids are deterrents to predators when systemic. In that direction, personal observations of Bowers [32], the chief investigator on this system, indicate that larvae of *Ce. catalpae* - which contain catalpol - are unpalatable to birds whilst adults - which do not retain catalpol - are palatable. Iridoids may also be utilized by insects in higher trophic levels, for instance, catalpol was found in low amounts in the larval parasitoid *Cotesia congregata* (Hymenoptera: Braconidae), a not a surprising fact considering that the chemical accounts for almost half of the dry weight of the *Ce. catalpae* larva hemolymph. The issue that remains unclear is whether *C. congregata *uses the catalpol acquired from its host, *Ce. catalpae*, as chemical defense. However, iridoid sequestration by higher trophic levels, such as parasitoids, is not the general rule. *Cotesia melitaearum*, a parasitoid of *Melitaea cinxia*, prefers the larvae with lower concentrations of catalpol [3]. This fact suggests that catalpol may be acting as an effective chemical defense directed to *M. cinxia *larvae, deterring oviposition by this parasitoid wasp.

### 2.3. Iridoids: Produced as Induced Defenses

One wonders whether *Ce. catalpae* may induce a higher iridoid production after plant attack similarly to the specialist *Euphydryas aurinia* (Lepidoptera: Nymphalidae), which after laying eggs on leaves of *Lonicera implexa* (Caprifoliaceae), lead to an over 15-fold increase of the amounts of iridoids [secoxyloganic acid (**4**), loganic acid (**5**) and secologanic acid (**6**)] [31]. As far as we know, there have been no studies addressing this issue. However, it is known that *Catalpa bignonioides* has the ability to produce induced responses after *Ce. catalpae* attack. This induction changes the interaction established in the association between, *C. bignonioides* and formica ants. When *C. bignonioides* is exposed to the sphingid attack, it produces more nectar in extrafloral nectarines located in the caterpillar-infected leaves. As ant´s attendance to these leaves (as well as the arrival of predators and parasitoids of *Ce. catalpae* larvae) increases, *Ce. catalpae *larvae are mechanically removed by the ant workers [27,33]. It is well documented that the mechanical damage produced by the larvae when feeding triggers the increased production of nectar [33], however further studies should address whether some chemical factors are also involved. Therefore, since *C. bignonioides* has the capacity to respond to the herbivore damage, and -as it is known- induced responses have central biochemical pathways ubiquitous to different kind of responses [34], it could be possible that iridoids may also be produced as induced response. 

### 2.4. Iridoids: Specialization, Tolerance and Addiction

Iridoids have been shown to be deterrents as well as phagostimulants towards different potential consumers. Well documented examples of specialization based on iridoids are the cases of two nymphalids: the chekerspot *Euphydryas chalcedona *(although *E. chalcedona *is not actually considered a complete specialist) [4], and the buckeye butterfly *Junonia coenia* [35]. Although none of them are specialists on Bignoniaceae, both species usually utilize plants from iridoid-containing families (*E. chalcedona *feeds on Scrophulariaceae, Plantaginaceae, Caprifoliaceae and Oleaceae; and *J. coenia *on Scrophulariaceae, Plantaginaceae, Verbenaceae and Acanthaceae). Phagostimulation by catalpol to *E. chalcedona *and by aucubin (**3**) [4] to *J. coenia* was evidenced [35]. Moreover, to *J. coenia*, diet iridoids are essential for its development, as their absence produces poor growth and lower survival, indicating a dependence on them that may mediate the specialist relationship with its food plant [35]. Being both catalpol and aucubin (**3**) ubiquitous iridoids within the Bignoniaceae, it may be possible that this dependence is also present in Bignoniaceae specialists. Obviously, specialist herbivores feeding on iridoid-containing plants find those secondary chemicals palatable. But at some point during evolution the same chemicals that were selected as defensive clues (*i.e.* antifeedants) to potential herbivores, had to be tolerated by the herbivore overcoming such defenses. With time the tolerance had to evolve to specialization linked to those chemicals that allowed the selection of the food source by the insect. Indeed, habituation to iridoids, the defensive chemicals of Bignoniaceae, was demonstrated at least for two polyphagous noctuids. Larvae of the southern armyworm (*Spodoptera eridania*) do not find iridoids in their usual food sources (herbaceous plants), subsequently, when they were presented with iridoid-enriched diets [catalpol, aucubin (**3**), loganin (**7**) or asperuloside (**8**)], the four iridoids arrested feeding. However, when larvae reared from hatching on these same diets were given a choice between diets with and without those iridoids, they did not show any preference for the control [36]. In this case, pre-exposure seems to conduct to habituation, eventhough larvae reared on diets containing iridoids grow more slowly (catalpol) or do not grow at all (aucubin and loganin). Analogous observations were done in the case of the gypsy moth larvae (*Lymantria dispar*) which -if preexposed to iridoids- chose to feed on an iridoid containing diet which caused them to have lower growth and survival [37]. Iridoids are not the only detrimental natural products that bring about this kind of addictive behavior that decreases fitness, for instance, nicotine holds the same effect on *Manduca sexta* (Lepidoptera: Sphingidae) larvae [38].

To sum up, the ecological interaction mediated by iridoids between the specialist *Ce. catalpae* and *Catalpa *spp.is complex. In *Catalpa*, iridoids possibly function as chemical defenses against other herbivores, and as kairomones towards *Ce. catalpae*. By the same token *Ce. catalpae* larvae are able to metabolize, sequester and presumably use those iridoids for their own defense against predators and/or parasitoids. These interactions provide a good example of one kind of chemical structure accomplishing different semiochemical roles. Although, some issues, such as the likely roles of iridoids as oviposition markers, inducted defenses and habituation mediators, deserve further studies yet. 

## 3. Bignoniaceae Secondary Metabolites with Anti-Insect Properties

### 3.1. Iridoids

Many iridoids, some coming from Bignoniaceae, some coming from other plants but present also in Bignoniaceae, were tested for their anti-insect properties against insects other than the ones mentioned in the previous sections. In the following cases, the insect species tested do not usually find iridoids in their usual diets in Nature. For instance, ipolamiide (**9**) presented strong anti-feedant properties towards three of the most important pest species in agriculture [*Locusta migratoria*, *Schistocerca gregaria* (Orthoptera: Acrididae) and *Spodoptera littoralis* (Lepidoptera: Noctuidae)] [39], and catalpol was deterrent against *Camponotus floridanus* (Hymenoptera: Formicidae) [40]. Choice tests with catalpol-enriched and catalpol-free sugar solutions were conducted evidencing ant preference to the catalpol-free diet. This deterrent effect of catalpol was dose dependent as solutions with low catalpol (**1**) concentration were consumed in non-choice experiments. However, in some cases isolated iridoids showed no effect, even if the iridoid extracts from which they come from do. That is the case of catalposide (**2**) and specioside (**10**) inactive by themselves against *Lymantria dispar* (Lepidoptera: Noctuidae) [41]. This observation may be accounted by the presence of other compounds in the original iridoid extract or by a synergistic effect between catalposide and specioside. However, in another study, catalposide by itself was not chosen when larvae were given the option of diets with lower concentrations, in a negative dose-response pattern [42].

In an effort to find a control agent against the tortricid *Choristoneura fumiferana*, the Eastern spruce budworm, non susceptible trees were prospected; leading to the isolation of a new iridoid named specionin (**11**) from *Catalpa speciosa *leaves with anti-feedant properties [43,44]. 

### 3.2. Naphtoquinones

**Figure 2:**
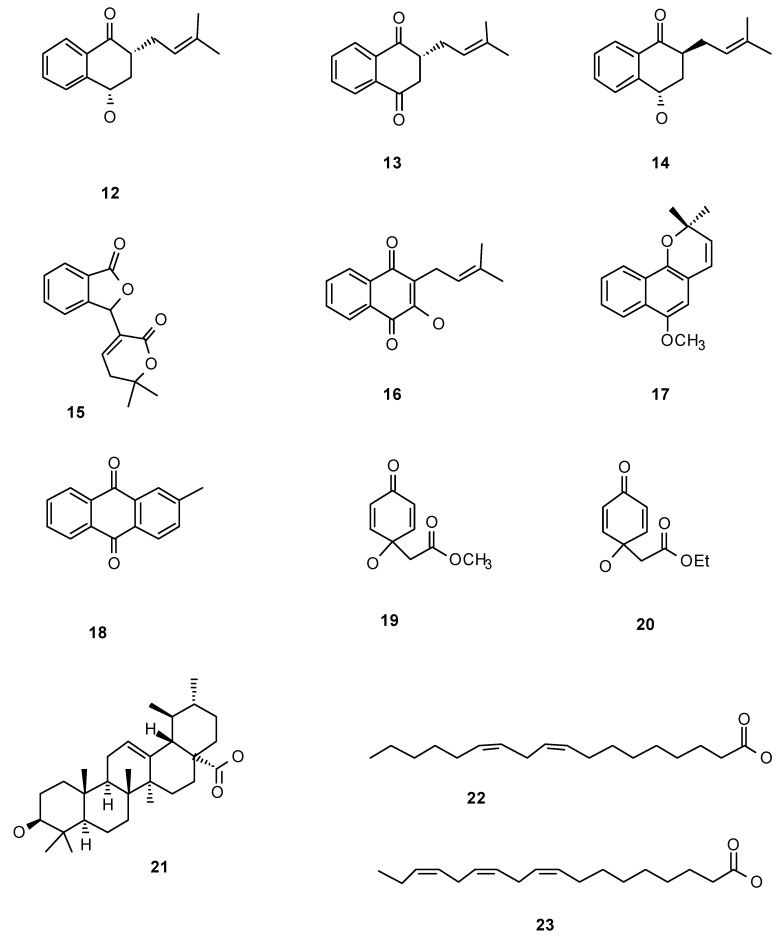
Naphthoquinones and miscellaneous chemicals.

Besides iridoids, extracts and other compounds isolated from this family were tested in their anti-insect properties (Table 1). The Bignoniaceae include several species which woods exhibit variable degrees of resistance against termite attack and therefore these species have been included in various prospecting programs seeking for the wood chemical characteristics that confer termite resistance. Naphthoquinones extracts isolated from *Catalpa bignonioides* showed activity against the termite *Reticulitermes flavipes* (Isoptera: Rhinotermitidae). Specifically, the most abundant quinones, catalponol (**12**) and catalponone (**13**) (Figure 2) when offered in experiments where a choice between control and treated wood pieces were offered, these termites did not choose at all the surfaces treated with the naphtoquinone extract. However, when there were no options available, *R. flavipes *utilized woods treated with a mixture of both naphthoquinones; and as a result, the termite survival decreased down to 0% in 7 days. The other, less abundant compounds, epicatalponol (**14**) and catalpalactone (**15**) presented a much lower toxicity [45]. It is noteworthy that stereoisomerism alters the activity as the epimers epicatalponol and catalponol show drastically different activity in both bioassays. Therefore the anti-termite extract from *C. bignonioides* contains a mixture of quinones with remarkable dual activity. Some of them constitute a first defensive line as they deter feeding, but if termites overcome somehow this deterrence, the quinones are toxic to them after consumption, illustrating the arms race established between these two species. Likewise, *Tabebuia ochraceae *-and *T. guayacan*- extracts repel *R. hesperus* on behavioral assays, but if consumed, decrease survival compared to starvation controls, indicating toxicity [46]. Isolated naphthoquinones from *Tabebuia* spp. such as lapachenole (**17**) and tectoquinone (**18**) also showed repellence by themselves against various *Reticulitermes *species (Rhinotermitidae), as well as against Termitidae and Kalotermitidae species. However this is not a general pattern, for instance lapachol (**16**) also isolated from *Tabebuia *spp. showed no repellent activity to *Reticulitermes* termites [but it was repellent to two other termites, *Microcerotermes crassus* (Isoptera: Termitidae) and *Kalotermes flavicollis* (Isoptera: Kalotermitidae)] [47]. These studies clearly illustrate two common facts in regard to antifeedant natural products: first, extracts (mixtures) seem to work better than isolated compounds (naphthoquinones here); and second anti-insect (termite) activity is not a general activity of a group of structurally related semiochemicals (naphthoquinones) as small changes in the molecules significantly change activity. Finally, different termite species idiosyncratically respond to these natural products [47].

Bignoniaceae extracts containing naphthoquinones are also active against Diptera species. Perhaps as a general pattern, the anti-Diptera activity may be tracked down to quinones [48]. Lapachol (**16**) isolated from *Cybistax antisyphilitica* wood extracts accounted for the extract activity against *Aedes aegypti* (Diptera: Culicidae) larvae. Larvicidal effect was quick: the LD_50_ concentration (26 mg/mL) was already active in 30 minutes [49]. Jacaranone (**19**), extracted from species of *Jacaranda*, one of the most known Bignoniaceae genus, showed activity against houseflies. In this case, jacaranone (**19**) was isolated from an Asteraceae (*Senecio palmatus*) when tested, but it was reported in many Bignoniaceae species [50]. Jacaranone (**19**) exhibited an effect depending upon the application mode: when topically treated houseflies were not affected, however, when offered as part of a sugar diet, it was toxic after ingestion [50]. Finally, in the case of the tobacco cutworm *Spodoptera litura *(Lepidoptera: Noctuidae), jacaranone (**19**) and its analogue (**20**) bearing an ethyl ester moiety instead of a methyl group were both deterrent on 3^rd^-instar larvae, and growth inhibitors of neonate larvae [51].

### 3.3. Other Secondary Metabolites: Miscellaneous Compounds and Whole Plant Extracts

Worth mention, a study on *Jacaranda decurrens *extracts with anti-aphid activity, led to the isolation of ursolic acid (**21**), a ubiquitous secondary metabolite in Nature, as the compound exhibiting that activity. The aphid *Schizaphis graminum* (Hemiptera: Aphididae), was not only deterred-ingestion times on ursolic acid enriched diets decreased compared to control diets- but also affected in their fitness. Survival, reproduction rate and population growth, were all negatively affected by ursolic acid in the diet, in a dose-dependent response [52].

The ethnobotanical and economic uses of Bignoniaceae extracts were previously covered quite comprehensively [7]. Concerning the uses against insects, the only mention this author made is the use of *Mansoa* sp. extracts as an insect repellent ([53] as cited by [7]). Other examples of whole extracts with activity are found in the cases of acetone extracts from leaves of *Millingtonia hortensis* which exhibited larvicidal effects against *Anopheles stephensi* (Diptera: Anhophelinae), *Culex quinquefasciatus* and *Aedes aegypti* (Diptera: Culicidae) [48]. *Dolichandra cynanchoides* aerial-part extract was tested for different activities, including feeding deterrence to *Epilachna paenulata* (Coleoptera: Coccinellidae) larvae [54]. Indeed during the course of a prospection program of local plant species, we have also tested extracts from this species against *E. paenulata*, but in our case we have used adults [55]. This screening on plants from several families searching for anti-insect activities included, besides *D. cynanchoides*, *Clytostoma callistegioides*, *Macfadyena unguis-cati* and *Jacaranda mimosifolia *(Table 2). These species belong to the tribe Bignonieae, with the exception of *J. mimosifolia *which is a member of the Tecomeae. Bignonieae in general have been reported as negative for the presence of iridoids, with the exception of *D. cynanchoides* [2]. Although *J. mimosifolia* has been reported as iridoid-free, it belongs to a tribe where the presence of iridoids formylated in C4 is characteristic [2]. In our studies, leaves were extracted by soxhlet with ethanol, and the crude extracts were tested in their anti-acari and anti-insect capacities.

**Table 1:** Extracts and compounds from Bignoniaceae with anti-insect or phagostimulant activities.

Plant species	Extract/ Compound	Insect	Activity	Ref.
*Catalpa bignonioides*	**12**,**13**	*Reticulitermes flavipes*	Toxic	[45]
*Catalpa speciosa*	**1**,**2**	*Ceratomia catalpae*	Phagostimulant	[27]
	**11**	*Choristoneura fumiferana*	Antifeedant	[43]
	**2**	*Camponotus floridanus*	Deterrent	[40]
*Clytostoma callistegioides*	Leaf extract	*Epilachna paenulata*	Deterrent	[55]
		*Myzus persicae *		
		*Rhopalosiphum padi*		
	**22**,**23**	*Myzus persicae*	Deterrent	[58]
*Cybistax antisyphilitica*	**16**	*Aedes aegypti*	Toxic	[60]
*Dolichandra cynanchoides*	Leaf extract	*Epilachna paenulata*	Deterrent	[54,55]
		*Myzus persicae*		
		*Rhopalosiphum padi*		
*Jacaranda decurrens*	**21**	*Schizaphis graminum*	Toxic/ Deterrent	[52]
*Jacaranda *sp.	**19**	*Musca domestica*	Toxic	[50]
		*Spodoptera litura*		[51]
*Macfadyena unguis-cati*	Leaf extract	*Epilachna paenulata*	Deterrent	[55]
		*Myzus persicae*		
		*Rhopalosiphum padi*		
*Millingtonia hortensis*	Leaf Extract	*Anopheles stephensi*	Toxic	[48]
		*Culex quinquefasciatus*		
		*Aedes aegypti*		
*Spathodea campanulata*	Flower mucilage	*Scaptotrigona postica*	Toxic	[61]
*Tabebuia guayacan*	Wood extract	*Reticulitermes Hesperus*	Toxic/ Repellent	[46]
	**16**	*Microcerotermes crassus*	Repellent	[47]
		*Kalotermes flavicollis*		
*Tabebuia ochracea*	Sawdust extract	*Reticulitermes Hesperus*	Toxic/ Repellent	[46]
*Tabebuia sp*	**17**	*Reticulitermes sp*	Repellent	[47]

**Table 2:** Bioassay results for the different anti-arthropod activities in plant extracts from Uruguayan native Bignoniaceae.

Extract (organ extracted)	*M. persicae* ^1^	*R. * *padi * ^1^	*S. littoralis* ^2^	*E. paenulata* ^2^	*A. mellifera* ^3^	*R. * *microplus* ^ 4*^
*C. callistegioides* (Leaves)	Deterrent	Deterrent	Inactive	Deterrent	Innocuous	Inactive
*C. callistegioides* (Vines) *	Deterrent	Inactive	Inactive	Deterrent	NT	Inactive
*M. unguis-cati* (Leaves)	Deterrent	Inactive	Inactive	Deterrent	Innocuous	Inactive
*M. unguis- cati* (Vines) ^*^	Inactive	Inactive	Inactive	Deterrent	NT	Inactive
*D. cynanchoides* (Leaves)	Deterrent	Inactive	Inactive	Deterrent	Innocuous	Toxic
*J. mimosifolia* (Leaves) ^*^	Inactive	Inactive	Inactive	Deterrent	NT	Inactive

NT: not tested; ^1^ Deterrent means the extract inhibits settling of the insects compared to the control; ^2^ Deterrent means the extract inhibits feeding of the insects compared to the control; ^3^ Innocuous means the extract does not affect bee behaviour or survival compared to the control; ^4^ Toxic means fecundity index (oviposition and hatching rate) diminishes compared to the control; ^*^ Unpublished data, all other data are from [55].

The common cattle tick *Rhipicephalus (Boophilus) microplus * (Arachnida: Ixodidae) was used following an immersion test protocol [56] to search for activity. In this case, among the Bignoniaceae extracts tested (Table 2), only *D. cynanchoides* (leaf extract) showed an effect on fitness. Even though topical application (immersion) of the extract did not diminish oviposition, larval hatching rate decreased in a magnitude that made the fecundity index significantly lower than the one coming from the control (unpublished data).

Anti-insect activity was tested on leaf surfaces applying the extracts in amounts of 100 µg/cm^2^. The extracts were evaluated in their deterrence activity against four species of insects, adults of *E. paenulata*, larvae of *Spodoptera littoralis* (Lepidoptera: Noctuidae), and apterous adults of *Myzus persicae* and *Rhopalosiphum padi* (Hemiptera: Aphididae). The insect species used as models were selected according to their feeding habits and diet breadth in order to have a broader spectrum of the anti-insect activities. *E. paenulata* is a chewer specialist on Cucurbitaceae; *S. littoralis* a generalist chewer; and in the case of the piercing insects, *R. padi* is a grass specialist and *M. persicae* is a generalist. In addition, honeybees, as beneficial insects not to be damaged when looking for anti-insect products to be potentially included in botanical pesticide formulations, were included in this screening. The extracts showed innocuousness by contact to honeybees (Table 2). In the case of the former four insects, choice tests were performed offering an extract-treated together with a solvent-treated leaf surface. Anti-settling and anti-feeding activities were measured for the aphids and the chewer insects respectively. In the case of *M. persicae* most of the extracts proved to have anti-settling activity, while in the case of *R. padi*, only the extract of *C. callistegioides* displayed this capacity. For the chewer insects, none of the extracts was a feeding deterrent to *S. littoralis *larvae, while all of them presented that activity against *E. paenulata* adults [55] and unpublished data. This was the first report of the activity of these extracts against insects, with the exception of the extract from *D. cynanchoides*, whose leaf extract was also deterrent to *E. paenulata* larvae [54], although at higher concentrations (200 µg/cm^2^) than the one tested by us. Further studies were pursued with the most active extract, the one coming from *C. callistegioides* leaves (Table 2). Bio-guided fractionation monitored by the model *M. persicae* was completed to track down the biological activity which turned out to be based on a mixture of four fatty acids (palmitic, stearic, linoleic and linolenic acids). Tests on isolated fatty acids showed that the activity originates from linoleic (**22**) and linolenic (**23**) acids. The fact that such ubiquitous chemicals were settling inhibitors of aphids intrigued us. The amount of fatty acids - quantified by GC/MS - in waxes coming from young leaves was greater than the one from old leaves; being the younger leaves also richer in unsaturated fatty acids (the ones active). This result may be explained by the fact that younger leaves are preferred by aphids [57], and therefore they need to produce more deterrent defenses. On a lateral remark, as a part of this study, trichomes in both, adaxial and abaxial leaf surfaces were described for the first time in *C. callistegioides*. Those trichomes stained for the presence of total and unsaturated lipids stronger than in the rest of the leaf surface, probably indicating that the fatty acids may be concentrated in and liberated from them [58]. Recent studies allowed isolating other active compounds from *C. callistegioides *leaf extracts. The aglycone fraction of its glycosides presents comparable anti-insect activities than the crude ethanolic extract. After HPLC purification, the main compounds in this fraction were characterized mostly as flavonoids which when individually tested against the two aphids, showed differential activities (unpublished data). These data suggests that flavonoids may also be implied in the defense mechanisms of *C. callistegioides*.

From these studies, it may be concluded, that iridoids are the more studied compounds in the family, not only in terms of feeding deterrence, but also in terms of semiochemical signals used by specialist insects to find their oviposition sites and food sources. In these line, as deterrents, iridoids can be considered allomones for the Bignoniaceae species that biosynthesize and use them as defenses; in contrast when iridoids are oviposition or feeding stimulants to specialists, they constitute kairomones since their presence rather than being advantageous to the plant, benefits their herbivores. The other big group of compounds present in Bignoniaceae with anti-insect properties are de quinones (naphtho- and anthraquinones mainly). For quinones, studies are scarcer, having been characterized as semiochemicals clues only in the case of termites (all other studies deal with potential botanical pesticides). Clearly more studies are needed to elucidate the herbivorous-plant interactions in systems where Bignoniaceae participate. At any rate, Bignoniaceae semiochemicals have also proved to be antifeedant or toxic agents against various species belonging to the insect orders where most of the pests are found (Coleoptera, Diptera, Hemiptera, Hymenoptera, Lepidoptera and Orthoptera). Up to now, iridoids and naphthoquinones have been shown to be the main active principles in this sense, although more recent studies are evidencing that metabolites such as triterpenes and flavonoids may also exhibit anti-insect activity. All the anti-insect properties so far reported, the structural diversity of secondary metabolites from Bignoniaceae, and the fact that this family is not among the classical ones when prospecting for potential botanical pesticides [59] may point to this family as a promising source of anti-insect agents. 

## References

[1] Gentry A. (1980). Bignoniaceae—Part I (Crescentiae and Tourretaeae). Flora Neotropica, 25.

[2] Von Poser G.L., Schripsema J., Henriques A.T., Jensen S.R. (2000). The distribution of iridoids in Bignoniaceae. Biochem. Syst. Ecol..

[3] Nieminen M., Suomi J., Van Nouhuys S., Sauri P., Riekkola L. (2003). Effect of iridoid glycoside content on oviposition host plant choice and parasitism in a specialist herbivore. J. Chem. Ecol..

[4] Bowers D. (1983). The role of iridoid glycosides in host-plant specificity of checkerspot butterflies. J. Chem. Ecol..

[5] Bowers D. (2003). Hostplant suitability and defensive chemistry of the catalpa sphinx, *Ceratomia catalpae*. J. Chem. Ecol..

[6] Lozoya-Meckes M., Mellado-Campos V. (1985). Is the *Tecoma stans* infusion an antidiabetic remedy?. J. Ethnopharmacol..

[7] Gentry A. (1992). A synopsis of bignoniaceae ethnobotany and economic botany. Ann. Mo. Bot. Gard..

[8] Aguilar-Santamaria L., Ramirez G., Nicasio P., Alegria-Reyes C., Herrera-Arellano A. (2009). Antidiabetic activities of *Tecoma stans* (L.) Juss. ex Kunth. J. Ethnopharmacol..

[9] Barbosa J., Lima C., Amorin E., de Sena K., Almeida J., da cunha E., Silva M., de Fitima M., Braz R. (2004). Botanical study, phytochemistry and antimicrobial activity of *Tabebuia aurea*. Phyton (B. Aires).

[10] Kim D., Han K., Chung L., Kim D., Kim S., Kwon B., Jeong T., Park M., Ahn E., Baek N. (2005). Triterpenoids from the flower of *Campsis grandiflora* K. Schum. as human Acyl-Cok cholesterol acyltransferase inhibitors. Arch. Pharmacol. Res..

[11] Ali R.M., Houghton P.J., Hoo T.S. (1998). Antifungal activity of some Bignoniaceae found in Malaysia. Phytother. Res..

[12] Alcerito T., Barbo F.E., Negri G., Santos D.Y.A.C., Meda C.I., Young M.C.M., Chavez D., Blatt C.T.T. (2002). Foliar epicuticular wax of Arrabidaea brachypoda: flavonoids and antifungal activity. Biochem. Syst. Ecol..

[13] Lima C.S.D.A., Cavalcanti de Amorim E.L., Xiato da Fonseca K., de Sena R., Chiappeta A.d.A., Nunes X.P., Agra M.D.F., Leitao da-Cunha E.V., Sobral da Silva M., Barbosa-Filho J.M. (2003). Antimicrobial activity of a mixture of two isomeric phenylpropanoid glycosides from *Arrabidaea harleyi* A.H. Gentry (Bignoniaceae). Rev. Bras. Cienc. Farm..

[14] Onegi B., Kraft C., Kohler I., Freund M., Jenett-Siems K., Siems K., Beyer G., Melzig Matthias F., Bienzle U., Eich E. (2002). Antiplasmodial activity of naphthoquinones and one anthraquinone from *Stereospermum kunthianum*. Phytochemistry.

[15] Reis de Morais S.K., Silva S.G., Portela C.N., Nunomura S.M., Quignard E.L.J., Pohlit A.M. (2007). Bioactive dihydroxyfuranonaphthoquinones from the bark of *Tabebuia incana* A.H. Gentry (Bignoniaceae) and HPLC analysis of commercial Pau d' Arco and certified *T. incana* bark infusions. Acta Amazonica.

[16] Hussain H., Krohn K., Ahmad V.U., Miana G.A., Green I.R. (2007). Lapachol: An overview. ARKIVOC.

[17] Ventura Pinto A., Lisboa de Castro S. (2009). The trypanocidal activity of naphthoquinones: a review. Molecules.

[18] Lima N., Correia C., Leon L., Machado G., Madeira M., Santana A., Goulart M. (2004). Antileishmanial activity of lapachol analogues. Mem. Inst. Oswaldo Cruz.

[19] Erlich P.R., Raven P.H. (1964). Butterflies and plants: A study in coevolution. Evolution.

[20] Eisner T., Meinwald J. (1995). Chemical Ecology. Proc. Natl. Acad. Sci. USA.

[21] Meinwald L., Eisner T. (1995). The chemistry of phyletic dominance. Proc. Natl. Acad. Sci. USA.

[22] Bergström G. (2007). Chemical ecology = chemistry + ecology!. Pure Appl. Chem..

[23] Karlson P., Butenandt A. (1957). Pheromones (ectohormones) in insects. Annu. Rev. Entomol..

[24] Bowers D., Rosenthal G., Berenbaum M. (1991). Iridoid glycosides.

[25] Hodar J. (2002). Host utilisation by moth and larval survival of pine processionary caterpillar Thaumetopoea pityocampa in relation to food quality in three Pinus species. Ecol. Entomol..

[26] Larsson S., Ekbom B. (1995). Oviposition mistakes in herbivorous insects: confusion or a step towards a new host plant. Oikos.

[27] Stephenson A. (1982). Iridoid glycosides in the nectar of *Catalpa speciosa* are unpalatable to nectar thieves. J. Chem. Ecol..

[28] Stephenson A., Thomas W.W. (1977). Diurnal and nocturnal pollination of *Catalpa speciosa* (Bignoniaceae). Syst. Bot..

[29] Stephenson A. (1982). The role of extrafloral nectaries of *Catalpa speciosa* in limiting herbivory and increasing fruit production. Ecology (NY).

[30] Pereyra P., Bowers D. (1988). Iridoid glycosides as oviposition stimulants for the buckeye butterfly, *Junonia coenia *(Nymphalidae). J. Chem. Ecol..

[31] Pañuelas J., Sardans J., Stefanescu C., Parella T., Filella I. (2006). *Lonicera implexa* leaves bearing naturally laid eggs of the specialist herbivore* Euphydryas aurinia* have dramatically greater concentrations of iridoid glycosides than other leaves. J. Chem. Ecol..

[32] Bowers D., Puttick G. (1986). Fate of ingested iridoid glycosides in lepidopteran herbivores. J. Chem. Ecol..

[33] Ness J. (2003). *Catalpa bignonioides* alter extrafloral nectar production after herbivory and attracts ant bodyguards. Oecologia.

[34] Howe G., Jander G. (2008). Plant immunity to insect herbivores. Annu. Rev. Plant Biol..

[35] Bowers D. (1984). Iridoids glycosides and host-plant specificity in larvae of the buckeye butterfly, *Junonia coenia* (Nymphalidae). J. Chem. Ecol..

[36] Puttick G., Bowers D. (1988). Effect of qualitative and quantitative variation in allelochemicals on a generalist insect: iridoids glycosides and the southern armyworm. J. Chem. Ecol..

[37] Bowers D., Puttick G. (1988). Response of generalist and specialist insects to qualitative allelochemical variation. J. Chem. Ecol..

[38] del Campo M., Miles C., Schoroeder F., Mueller C., Booker R., Renwick A. (2001). Host recognition by tobacco hornworm is mediated by a host plant compound. Nature.

[39] Bernays E., De Luca C. (1981). Insect antifeedant properties of an iridoid glycoside: ipolamiide. Experientia.

[40] de la Fuente M., Dyer L., Bowers D. (1994). The iridoid glycoside, catalpol, as a deterrent to the predator *Camponotus floridanus* (Formicidae). Chemoecology.

[41] El-Naggar S., Doskotch R. (1980). Speciocide: a new iridoid glycoside from *Catalpa speciosa*. J. Nat. Prod..

[42] Bowers D., Puttick G. (1989). Iridoid glycosides and insect feeding preferences: gypsy moths (*Lymantria dispar*, Lymantriidae) and buckeyes (*Junonia coenia*, Nymphalidae). Ecol. Entomol..

[43] Chang C., Nakanishi K. (1983). Specionin, an iridoid insect antifeedant from *Catalpa speciosa*. J. Chem. Soc. Chem. Commun..

[44] Van der Eycken E., Van der Eychen J., Vandewalle M. (1985). Iridoids: The revised structure of specionin. J. Chem. Soc. Chem. Commun..

[45] McDaniel C. (1992). Major antitermitic components of the heartwood of southern catalpa. J. Chem. Ecol..

[46] Grace K., Wood D., Frankie G. (1989). Behavior and survival of *Reticulitermes hesperus* banks (Isoptera: Rhinotermitidae) on selected sawdusts and wood extracts. J. Chem. Ecol..

[47] Becker G., Lenz M., Dietz S. (1972). Unterschiede im Verhalten und der Giftempfindlichkeit verschiedener Termiten-Arten gegenuber einigen Kernholzstoffen. Z. Angew. Entomol..

[48] Kaushik R., Saini P. (2008). Larvicidal activity of leaf extract of *Millingtonia hortensis* (Family: Bignoniaceae) against *Anopheles stephensi*, *Culex quinquefasciatus* and *Aedes aegypti*. J. Vector Borne Dis..

[49] Rodrigues A.M.S., de Paula J.E., Roblot F., Fournet A., Espindola L.S. (2005). Larvicidal activity of* Cybistax antisyphilitica* against *Aedes aegypti* larvae. Fitoterapia.

[50] Xu H., Zhang N., Casida J. (2003). Insecticides in Chinese medicinal plants: survey leading to jacaranone, a neurotoxicant and glutathione-reactive quinol. J. Agric. Food Chem..

[51] Lajide L., Escoubas P., Mizutani J. (1996). Cyclohexadienones-insect growth inhibitors from foliar surfaces and tissue extracts of *Senecio cannabifolius*. Experientia.

[52] Varanda E., Zuniga G., Salatino A., Roque N., Corcuera L. (1992). Effect of ursolic acid from epicuticular waxes of *Jacaranda decurrens* on *Schizaphis graminum*. J. Nat. Prod..

[53] Lewis W.H., Elvin-Lewis M., Gnerre M.C., Leeuwenberg A.J. (1987). Introduction to the ethnobotanical pharmacopeia of the Amazonian Jivaro of Peru. Medicinal and Poisonous Plants of the Tropics.

[54] Palacios S., Maggi M., Bazán C., Carpinella C., Turco M., Muñoz A., Alonso R., Nuñez C., Cantero J., Defago M., Ferrayoli C., Valladares G. (2007). Screening of argentinian plants for pesticide activities. Fitoterapia.

[55] Castillo L., González-Coloma A., Gonález A., Díaz M., Santos E., Alonso-Paz E., Bassagoda M., Rossini C. (2009). Screening of Uruguayan plants for deterrent activity against insects. Ind. Crops Prod..

[56] Drummond R., Ernst S., Trevino J., Gladney W., Graham O. (1973). *Boophilus annulatus* and *Boophilus microplus*: Laboratory test of insecticides. J. Econ. Entomol..

[57] Smith M., Rechcigl J.E., Rechcigl N.A. (1998). Plant resistance to insects. Biological and Biotechnological Control of Pests.

[58] Castillo L., Díaz M., González-Coloma A., González A., Alonso-Paz E., Bassagoda M., Rossini C. (2010). *Clytostoma callistegioides* (Bignoniaceae) wax extract with activity on aphid settling. Phytochemistry.

[59] Arnarson J.T., Durst T., Philogene B.J.R., Regnault-Roger C., Philogene B.J.R., Vincent C. (2005). Phytochemical discovery of new botanical insecticides. Biopesticides of Plant Origin.

[60] Rodrigues A., de Paula J., Roblot F., Fournet A., Espindola L. (2005). Larvicidal activity of *Cybistax antisyphilitica *against *Aedes aegypti* larvae. Fitoterapia.

[61] Trigo J., Santos W. (1999). Insect mortality in *Spathodea campanulata* (Bignoniaceae) flowers. Rev. Bras. Biol..

